# Botulinum Toxin Therapy for Psychiatric Disorders in Clinical Practice: A Retrospective Case Study

**DOI:** 10.3390/toxins15060385

**Published:** 2023-06-07

**Authors:** Franziska Lehnert, Insa Neumann, Tillmann H. C. Krüger, Marc A. Wollmer

**Affiliations:** 1 Asklepios Clinic North-Ochsenzoll, Asklepios Campus Hamburg, Medical Faculty, Semmelweis University, 22419 Hamburg, Germany; franziska.lehnert@semmelweis-hamburg.de (F.L.); insa.neumann@semmelweis-hamburg.de (I.N.); 2 Center for Systems Neuroscience, 30559 Hannover, Germany; krueger.tillmann@mh-hannover.de; 3 Department of Psychiatry, Social Psychiatry and Psychotherapy, Division of Clinical Psychology and Sexual Medicine, Hannover Medical School, 30625 Hannover, Germany

**Keywords:** botulinum toxin, depression, anxiety, mental disorder, psychiatry, outpatient

## Abstract

Inhibiting the facial expression of negative emotions via botulinum toxin A (BTX) has been shown to mitigate symptoms of clinical depression in randomized controlled trials. This retrospective case study sought to reproduce the beneficial effects of BTX in a naturalistic setting for major depressive disorder and collect casuistic data on its effect on other mental disorders. Moreover, we describe symptom development across multiple treatment cycles with BTX, and assess the implementation of additional injection targets in the lower face region. Participants were *N* = 51 adult psychiatric outpatients mainly seeking treatment for depression. Over 50% suffered from comorbid psychiatric conditions, predominantly generalized anxiety disorder (GAD) or borderline personality disorder (BPD). A pre–post case series design was adapted. All participants received BTX-injections in the glabellar region on at least one occasion. Some received additional injections in the mouth region and over multiple treatment cycles. Treatment response was followed up by self-rated scales at varying time intervals post treatment. The results showed that BTX may yield favorable outcomes across multiple and comorbid mental disorders, especially, however, for patients suffering from depression. It potentially prevents the recurrence of clinical symptoms if applied regularly. Adding additional regions of the face does not seem to be superior over applying it to the glabellar region alone. The results add to the growing evidence that BTX therapy is effective in alleviating symptoms of depression. Positive effects can be sustained and reinstated, when applied over multiple treatment cycles. Observed symptom reduction in other psychiatric disorders was less pronounced. Further research is needed to understand the mechanisms by which BTX therapy reduces psychiatric symptoms.

## 1. Introduction

Botulinum toxin (BTX) injections into the “frown muscles” of the glabellar region have repeatedly been shown to improve emotional well-being and reduce symptoms of clinical depression. The first antidepressant effects were reported in a case series by Finzi and Wasserman [1] and later corroborated in a series of randomized-controlled trials (RCTs) [2,3,4,5,6,7]. The results were further substantiated by meta-analyses and population scale data, supporting the efficacy and safety of this treatment [8,9,10]. While the mechanism of the BTX–mood relationship remains the subject of ongoing research, it is plausible that facial feedback mechanisms may play a pivotal role in the treatment’s success. The facial feedback hypothesis subsumes an extensive body of psychological research supporting the notion that facial expressions influence emotive states [11]. The hypothesis predicts that facial expressions initiate and modulate emotional experience. Hence, expressing an emotion facially will intensify it, while inhibiting this expression will reduce the experience of the respective emotion. This assumption was, in fact, already postulated by Charles Darwin, who found “the free expression by outward signs of an emotion intensifies it. On the other hand, the repression as far as possible, of all outward signs, softens out emotions” [12]. Due to its inhibitory effects on muscle contraction, BTX injections offer an effective paradigm to analyze the effects of facial inhibition on emotional experience.

To date, clinical evidence of efficacy is limited to major depressive disorder (MDD) and borderline personality disorder (BPD) [13]. However, since negative emotional states are eminent in most mental disorders, BTX therapy has transdiagnostic therapeutic potential across a variety of mental health conditions.

Since negative emotions are primarily expressed by a furrowed brow via contraction of the procerus and corrugator muscles, prior experiments [14,15,16], as well as the aforementioned RCTs, typically focused on the prevention of frowning. Facial electromyogram (fEMG) studies have confirmed that healthy and depressed individuals produce different patterns of facial muscle activity [17]. Generally, happiness was associated with decreased activity in the corrugator muscle and increased activity in the depressor anguli oris muscle. Sadness, however, appeared to produce a pattern of increased corrugator and depressor anguli oris activity. In contrast to healthy participants, depressed participants produced similar activation patterns in response to sad and angry imagery, but differed significantly in response to happy imagery: those with depression showed no decrease in corrugator activity. It thus appears that the lack of relaxation of the corrugator muscle differentiates depressed from nondepressed individuals. In addition, increased activity in the region of the mouth, as measured by depressor muscle activity, was associated with happiness, sadness, and particularly anger.

Research also indicates higher baseline corrugator muscle activity in depressed patients compared to healthy controls [18,19] and that elevated levels of corrugator electromyographie (EMG) were predictive of a successful treatment [20,21,22]. Larsen et al. [23] compared electromyographic activity of corrugator muscles activity with other muscles of the upper part of the face, specifically, the zygomaticus major muscle during positive and negative affective states. Consistent with the previously mentioned research, this study showed that valence had a consistently stronger effect on the corrugator supercilii muscle than on the zygomaticus major muscle, again confirming the former as a key player of the upper face in the expression of negative emotions. However, Keltner et al. [24] identified muscles of the lower face as playing a crucial role in the expression of negative emotions, as well. They showed that in addition to the activation of the aforementioned muscles, sadness is also expressed via contraction of the depressor anguli oris and mentalis muscle, responsible for pulling down the corners of the mouth and producing chin dimples, respectively.

As of now, RCTs have tested single facial BTX injections into the glabellar region (i.e., procerus and corrugator muscles) in predominantly female samples with unipolar depression [5] and borderline personality disorder [13]. The aim of the present case series was to expand on research supporting the beneficial effects of BTX by reporting results for depression, generalized anxiety disorder, and borderline personality disorder. Furthermore, we sought to observe and describe symptom development after multiple treatment cycles with BTX, and to assess the feasibility of adding treatment targets in the lower face region, specifically, the depressor anguli oris muscle and the mentalis muscle. By this, we hope to inform future treatment protocols and advance research on BTX in the treatment of mental disorders.

## 2. Results

### 2.1. Participants

Data from *N* = 51 consecutive psychiatric outpatients (16 male, 35 female) receiving BTX treatment were collected from patient files. A total of *N* = 8 (4 male, 4 female) patients were excluded from further analysis due to lack of follow-up data. The baseline sample characteristics of all analyzed patients are displayed in Table 1. Except for 1 patient, all had a diagnosis of depression; of these, *N* = 24 (55.81%) were diagnosed with a comorbid mental disorder (see Table 1). One patient had a diagnosed BPD, but no psychiatric comorbidity. All participants gave written informed consent before BTX treatment. Despite the retrospective study design, the local ethical committee was informed and granted approval.

Outcomes will be reported separately for each of the three self-assessment tools used.

None of the patients showed obvious or spontaneously reported significant side effects or harm after injection of BTX. The treatment led to a complete relaxation of the muscles of the glabellar region. On the other hand, innervation of the muscles of the perioral region was only partially inhibited to prevent an impairment of adequate lip closure.

### 2.2. BDI

After treatment with BTX, participants demonstrated a score reduction in cycle 1 (c1) of −9.00 points (*SD* = 11.00) and −10.67 points (*SD* = 11.86) at t1 (*N* = 33) and t2 (*N* = 24), respectively. The t-test confirmed this trend by showing a significant pre–post treatment change, t(35) = 4.85, *p* < 0.001, *d* = 0.808. At c1t1, 9 participants (26.47%) had partially responded, 3 (8.82%) had responded, and 6 (17.65%) had gone into remission. Hence, 18 (52.94%) of the 34 participants had benefited from the initial BTX injection, while 15 participants (44.12%) were categorized as “nonresponders”.

In the second treatment cycle (c2), participants started from a generally lower symptom severity level compared to the first cycle. In fact, baseline scores resembled the post-treatment score mean of c1 (see Table 2). Furthermore, we observed a continued reduction in BDI scores at c2t1 (*N* = 14) of −7.50 (*SD* = 10.03) and at c2t2 (*N* = 15) of −7.00 (*SD* =11.40). The pre- versus post-comparison showed that the reduction in c2 was also significant, t(20) = 3.25, *p* = 0.004, *d* = 0.710, with an average post-treatment score reduction of −6.83 (*SD* = 9.63). If contrasted with initial baseline BDI scores in c1, we found that at c2t2, after completing 2 cycles of treatment, 7 of the remaining 15 participants had entered remission (46.67%), 1 had responded (6.67%), and 3 had partially responded (20%). In 4 cases, we saw a nonresponse (26.67%).

At the baseline of the third cycle (c3t0), the inspection of the spaghetti plot demonstrates a rebound of BDI scores, which again attenuates after treatment with BTX. A similar, yet less pronounced, spike in symptom severity was observed between each cycle, including cycles 4 (c4) and 5 (c5). Due to the small sample size, no inferential tests were performed for c3 to c5. Notably, values did not return to the level of the initial t1t0 baseline, indicating a sustained effect over multiple cycles.

### 2.3. BSL-23

A marked reduction in score could be found on the BSL-23, as well. In the pre–post comparison, patients showed significantly higher scores at c1t0 (*M* = 42.77, *SD* = 19.53) compared to after the treatment (*M* = 30.69, *SD* = 12.81), t(12) = 2.22, *p* = 0.046, *d* = 0.617 and an overall score reduction of 28.25%. As does the BDI, the spaghetti plot shows the most profound spike in scores at the baseline of cycle 3 (c3t0), with a marked decline after BTX treatment. While score levels increased with time after each treatment, the overall decrease in scores over time implies a continued effect over repeated treatment cycles for symptoms of BPD, as well. For the remaining cycles, sample sizes had become too small to continue further inferential analyses.

### 2.4. GAD-7

Nine patients of our sample suffered from comorbid GAD and were handed the GAD-7. The mean score of this sample at c1t0 was 15.00 (*SD* = 3.57). At t2, two patients had dropped out. While a slight reduction in score had occurred at all c1 follow-ups (c1t1: *M* = 14.22, *SD* = 3.46; c2t2: *M* = 13.00, *SD* = 4.58), the trend appears weak and the sample size too small to compute further analyses.

### 2.5. Comparison of Treatment Locations

**Figure 1:**
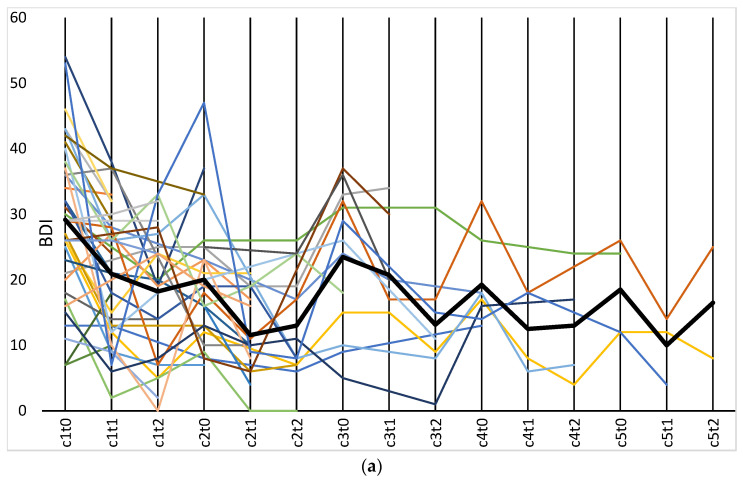

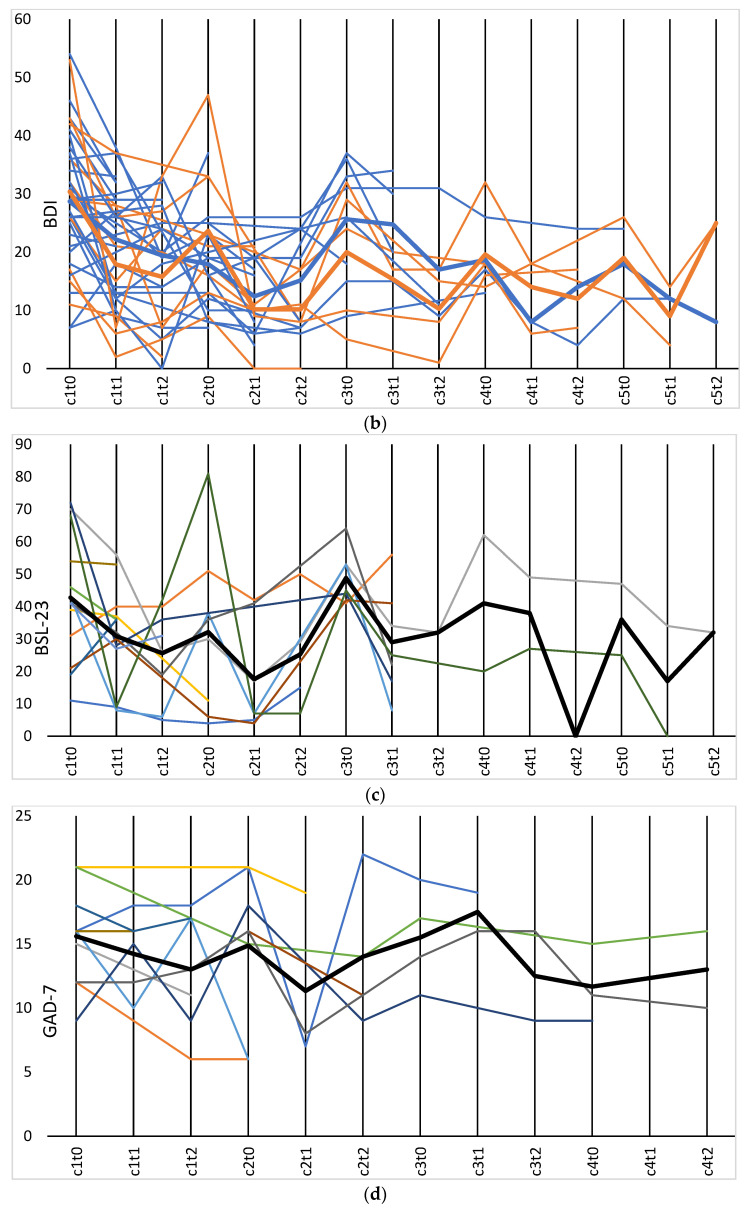
(**a**) BDI data displayed as spaghetti plot (*N* = 36). The bold line represents the sample mean. (**b**) BDI data displayed as spaghetti plot with distinction between upper face treatment only (blue, *N* = 26) and upper and lower face treatment (orange, *N* = 10). The bold lines represent the sub-sample means. (**c**) BSL-23 displayed as spaghetti plot (*N* = 13). The bold line represents the sample mean. (**d**) GAD-7 displayed as spaghetti plot (*N* = 11). The bold line represents the sample mean.

To analyze whether additional BTX injections in the region of the chin and mouth, specifically, to the m. mentalis and m. depressor anguli oris, would yield additional antidepressant effects, a binary dummy variable coding upper face treatment only (UF) vs. upper and lower face treatment (ULF) was added to the model as a between-subjects variable. The main effect of treatment was confirmed F(1,31) = 23.33, *p* < 0.001, ηp2= 0.429; however, there was no significant interaction between treatment and additional lower face injections F(1, 31) = 1.47, *p* = 0.234, ηp2 = 0.045, suggesting no superiority of ULF treatment over glabellar treatment alone. As depicted in Figure 1b and Table 3, there is no clear trend or discernable difference between patients with UF and ULF over time.

Figure 1a–d. Spaghetti plots of the different outcome measures at baseline (t0) and follow-up (t1 and t2) during succeeding cycles. Each thin line, all drafted in different colors, represents one participant. The bold line(s) represent(s) the sample mean.

## 3. Discussion

This retrospective case series adds to the increasing evidence for an antidepressant effect of facial, specifically, glabellar, injections of botulinum toxin. It investigated the treatment in a naturalistic setting and is the first to report results of additional treatment sites in the region of the chin. Furthermore, the present study observed long-term effects after multiple treatment cycles and included patients with varying, oftentimes comorbid, mental disorders.

In line with previous research, a strong effect could be shown for depression, measured by the BDI. While the average patient of our sample was severely depressed prior to the first application of BTX (*M* = 29.6), a score reduction of 10.5 points had been achieved after the first treatment cycle, representing a transition from severe to moderate depression. Scores at the end of the study were even lower (cycle 5), defining the average level of depression as mild (see “data collection” for cut-off scores). Notably, average depression scores did, at no point, return to baseline levels, indicating a sustained effect over multiple treatment cycles.

Looking at the development in more detail, the incline in scores with increased time passed after injection, as well as the marked decline in scores after injection, suggests that injections positively influence the course of the mood disorder. This is in line with previous research showing antidepressant effects of glabellar BTX injections. The effect size of d = 0.808 falls within the “large” range, suggesting that BTX has a meaningful influence on depression.

Interestingly, there was a marked reemergence of depression symptoms, as indicated by the BDI, after two cycles of BTX treatment. Self-selection bias might serve as one possible explanation. While the majority benefited from one or two treatment cycles, a subgroup of patients, potentially those with more severe symptoms or chronic depression, experience a resurgence of symptoms, once the effects wear off, and continue seeking treatment, while those with successful treatment or mild symptoms no longer sought treatment at the out-patient clinic. Another explanation would be that treatment adherence declined over time, and that the time intervals between the injections increased, resulting in a rebound of depressive symptoms. Comparing the time intervals that patients, who completed at least 3 cycles, waited to return for a follow-up injection, a substantial increase in number of weeks could be observed (*M* = 20.86 weeks (*SD* = 13.01) between cycle 1 and 2, *M* = 31.4 weeks (*SD* = 24.45) between cycle 2 and 3).

Pre–post comparisons in the group of BPD patients indicated a significant improvement on the treatment. Again, remarkable is a spike in symptom severity at c3t0, with values even exceeding the c1 baseline. Similar possible explanations apply as described above for patients suffering from depression. In line with what could be described for the latter, continuous additional treatments replicated the clinical improvements over time.

Note, however, that the RCT conducted by Wollmer et al. did not find superiority of BTX in BPD patients compared to a minimal acupuncture control group [13].

Generalized anxiety disorder is another common condition associated with negative emotional states. However, investigation of the difference between baseline and follow-ups showed no significant decrease in GAD-7 scores over the course of multiple treatment cycles. Thus, these data do not suggest a beneficial effect of BTX injections in this indication.

Even though muscles of the upper, as well as lower, face are active when producing expressions of negative affect [17], our results suggest that the major benefit may lie in the treatment of muscles of the glabellar region. Additional treatment targets in the region of the lower face delivered no superior response. However, the partial inhibition of these muscles, in comparison to a complete inhibition of the muscles of the upper face, needs to be taken into account when comparing the effects.

The fluctuation in the courses of the disorders over time mentioned above leaves room for discussion on the sustainability of the effect of BTX injections. The present study, as the majority of studies in this research area, used a maximum time interval of 12 weeks, and variations of breaks between cycles were large. The sustained effect seemed to be dependent on treatment continuation.

Magid et al. [4] demonstrated that the antidepressant effect may sustain for a time period of at least 24 weeks, greatly exceeding the time intervals tested before, as well as exceeding cosmetic effects, shown to last 12–16 weeks. Further research is needed to determine optimal treatment intervals.

Besides the interruption of proprioceptive afferences from the face to the emotional brain (i.e., the facial feedback hypothesis), other additional or alternative mechanisms may explain the antidepressant effect of glabellar BTX injections. One of them is social feedback. If the facial expression is shifted from negative to positive, it may elicit a more positive social response from others, which may in turn have a positive effect on mood [25].

Cosmetic effect may improve self and body image and thereby enhance mood. However, observations from previous RCTs including reduction in depressive symptoms in the absence of a favorable cosmetic effect, antidepressant effects in patients without obvious frown lines, and antidepressant effects outlasting the reduction in frown line severity argue against a major role of esthetic benefits in mood improvement [3,4,5].

It is possible that a fraction of the injected BTX will undergo retrograde transport into the central nervous system, which implies that central pharmacological effects may theoretically be involved in the antidepressant effect [26].

There is evidence that glabellar injections of BTX may modulate the activity of the amygdala, a central structure in the processing of particularly negative emotions [27]. Modulation of amygdala activity may be a neuronal correlate of the antidepressant effect observed after BTX treatment.

The present study sought to describe multiple cases of BTX treatment. It was not designed to measure cause and effect relationships. Its main limitation lies in the lack of a control group, which makes it difficult to distinguish between specific treatment effects, placebo effects, and spontaneous changes in the symptomatology. Furthermore, as indicated above, self-selection bias might have led to the presentation of higher scores on the self-rating scales applied, as patients with no or mild symptoms after the first treatment might have not returned for further treatment.

The great strength of the study lies in its high ecological validity due to its naturalistic setting. Additionally, patients were followed up for a long period of time, revealing continued changes and fluctuations in symptom change after varying time intervals. Finally, the replication of time effects, regarding depression and borderline personality disorder, add to the existing literature, supporting the notion that regular injections of BTX into the glabellar region may have a beneficial effect in these mental disorders.

## 4. Conclusions

This case series supports the beneficial effects of BTX injections in patients with depression and borderline personality disorder, but not for generalized anxiety disorder. Additional treatment targets in the region of the lower face did not demonstrate a superior effect over treatment in the glabellar region alone. Long-term treatment reveals that the alleviating effects seem to wear off over time. However, continued treatment led to sustained symptom reduction. This treatment method was confirmed to be applicable in naturalistic outpatient settings. BTX proves to be a valid treatment option in the management of depression and possibly BPD. Due to the limitations mentioned, further research is needed to fully establish BTX as a treatment for these conditions.

## 5. Materials and Methods

### 5.1. Procedure

**Figure 2:**
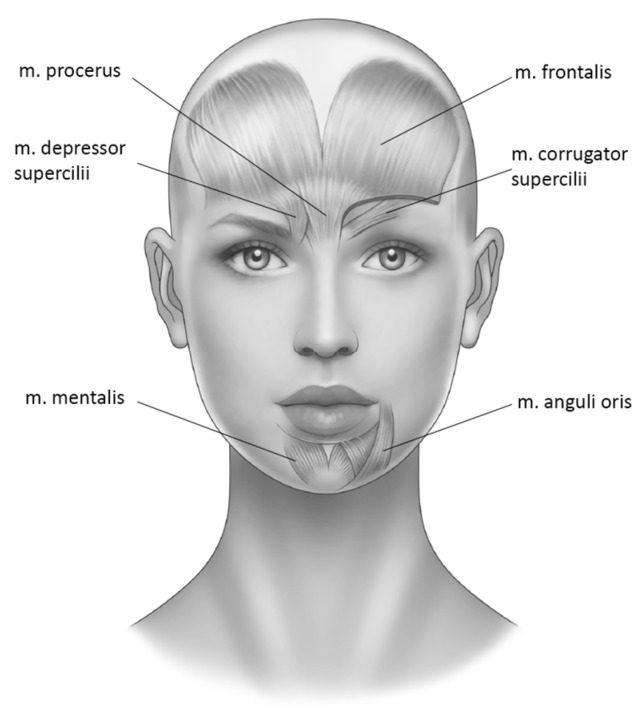
Depiction of the muscles injected with BTX (own representation).

Before the treatment, participants were informed about the reciprocal effect of facial expression and emotion. An explanation on how the permanent relaxation of muscles involved in the expression of negative emotions can alter the latter, was provided. All participants were treated with BTX at baseline (t0) and received a disorder-specific self-assessment questionnaire (see Section 5.2 and 5.4). Follow-up visits were typically planned after four and eight weeks post t0. However, there was considerable variation as to when participants came in for follow-up, which is typical for this kind of treatment setting. We defined two follow-up sessions, during which psychological assessment was performed. Some participants sent in the follow-up questionnaires via post. Some participants received multiple cycles of BTX treatment throughout the assessment period. Between each treatment cycle, there was a minimum three-month interval, since the paralyzing effect typically starts to wear off after that period. The treatment consisted of 23 to 47 units of onabotulinumtoxin A (Botox^®^ dissolved in 0.9% NaCl solution; 100 units/2.5 mL) injected with a 0.3 mL insulin syringe with 30-gauge needle. A few participants received incobotulinumtoxin A instead. The injection schemes varied among participants and were adjusted based on individual frown line visibility and muscle mass. Generally, concerning the muscles of the upper face, the sites treated were those commonly used in previous studies covering the use of BTX in the psychiatric field e.g., [14,15,16]. They are based on schemes used in esthetic medicine when treating frown lines. When treating participants who presented noticeable activation of the muscles of the perioral area, additional injections of BTX into the muscles deduced from the aforementioned paper by Keltner et al. [24] were provided. Units were distributed across 1 or more of the following target muscles: m. corrugator supercilii (*N* = 43, 100%), m. depressor supercilii (*N* = 29, 67.44%), m. procerus (*N* = 43, 100%), m. frontalis (*N* = 1, 2.33%), m. mentalis (*N* = 9, 20.93%), and m. depressor anguli oris (*N* = 7, 16.28%). See Figure 2 for a display of the treatment locations. Muscle relaxation was assessed by asking the participants to contract the eyebrows. If they were unable to perform this movement, muscle relaxation was assumed. The target muscles were identified using common anatomical knowledge. Points of injection varied among participants and were adjusted based on careful inspection and palpation of the muscles after asking patients to activate them. The procedure was performed by a trained physician (author MAW). The pychopharmacological or psychotherapeutical treatments were adjusted, if indicated.

### 5.2. Data Collection

Data were collected between August 2013 and September 2020 at an outpatient clinic in Hamburg, Germany and retrieved from patient files, which included standardized clinical questionnaires, medical reports, discharge letters, and medical notes. In order to assess the level of depression of our participants, we used the Beck Depression Inventory, a self-report inventory using 21 items, to assess the occurrence and severity of depressive symptoms over the course of the 2 weeks prior to the assessment [BDI]; [28]. By defining cut-off scores, the authors differentiated minimal (0–13), mild (14–19), moderate (20–28), and severe depression (29–63). Borderline personality disorder was assessed by the Borderline Symptom List-23, a self-rating questionnaire using 23 items to evaluate the level of severity of the borderline psychopathology in adults [BSL-23]; [29]. The severity of anxiety was rated using the Generalized Anxiety Disorder-7, a self-report questionnaire using seven items to evaluate how frequently symptoms of anxiety have appeared over the preceding two-week period [GAD-7]; [30].

### 5.3. Data Preparation

Since participants oftentimes received multiple treatments of BTX, data were analyzed by treatment cycle, where a treatment cycle was defined as an initial BTX treatment and baseline assessment (t0), with one (t1) or two (t2) follow-up assessments within a twelve-week period. Treatment cycles with missing pre- or post-treatment data were excluded. The t1 data subsume all scores within two to seven weeks post-treatment, and the t2 follow-up included all scores collected seven to twelve weeks post-treatment. No BTX treatments were performed within 12 weeks after t0, resulting in a minimum 12-week time interval between cycles. All other responses were discarded from the analysis because (a) the paralysis of BTX typically takes two weeks to fully come into effect, and (b) the paralysis starts to wear off after twelve weeks. For inferential statistical analyses, a mean across all post-assessments within each cycle was computed and entered as a single post-treatment score into the analysis, to reduce data loss.

### 5.4. Data Analysis

Descriptive statistics were displayed in tables and visualized in spaghetti plots in order to display the symptom change for every single case over the course of multiple treatment cycles (Figure 1a–d). Inferential statistical analyses were conducted on the BDI and the BSL-23 data to test for significant symptom change before and after treatment with botulinum toxin. Only the first cycles were analyzed due to loss of data as a consequence of dropout over time. Data were tested for normal distribution and analyzed using paired samples t-tests.

The alpha level was set at 0.05. Statistical analyses were performed using IBM SPSS Statistics version 28. Treatment response was defined as nonresponse (<25% reduction), partial response (25–49% reduction), response (>50% reduction), or remission (BDI score ≤ 10) at t2.

## Figures and Tables

**Table 1:** Sample characteristics at baseline (*N* = 43). All diagnoses classified according to ICD-10.

Variable	*N*	%	Range	*M*	*SD*
Gender					
Female	31	72.09			
Male	12	27.91			
Age (years)	43	100	29–80	51.28	12.76
Estimated time since initial diagnosis (years)	29	67.44	2–75	21.92	16.95
Duration of current depressive episode (months)	11	26.19	3–72	36.82	24.82
Primary diagnosis: Generalized Anxiety Disorder	1	2.33			
Primary diagnosis: Borderline Personality Disorder	5	11.63			
Primary diagnosis: Depression	37	86.05			
F31.3 Mild or Moderate Bipolar Depression	2	4.65			
F31.4 Severe Bipolar Depression	1	2.33			
F32.1 Moderate Depressive Episode	9	20.93			
F32.2 Severe Depressive Episode	9	20.93			
F33.1 Recurrent Depressive Disorder, Moderate	8	18.60			
F33.2 Recurrent Depressive Disorder, Severe	8	18.60			
Comorbid psychiatric diagnoses	24	55.81			
F11.2 Opioid Addiction	1	2.33			
F41.0 Panic Disorder	1	2.34			
F41.1 Generalized Anxiety Disorder	6	13.95			
F42.2 Obsessive–Compulsive Disorder	2	4.65			
F43.1 Post-Traumatic Stress Disorder	5	11.63			
F45.0 Somatization Disorder	1	2.33			
F50.2 Bulimia Nervosa	1	2.34			
F60.31 Borderline Personality Disorder	7	16.28			
Antidepressant medication	18	41.86			
Tricyclic	2	4.65			
SNRIs	5	11.63			
SSRIs	5	11.63			
MAOI	1	2.33.			
Others	5	11.63			
Other psychopharmaceuticals	29	67.44			
Antipsychotics	11	25.58			
Sedatives	4	9.33			
Lithium	4	9.33			
Others	10	23.26			
BDI	39	90.70	7–54	29.16	
BSL-23	13	30.23	11–27	40.77	
GAD-7	11	25.58	9–21	15.60	

SNRIs = serotonin and norepinephrine reuptake inhibitors; SSRIs = selective serotonin reuptake inhibitors; MAOI = monoamine oxidase inhibitors; BDI = Beck Depression Inventory; BSL-23 = Borderline Symptom List-23; GAD-7 = Generalized Anxiety Disorder-7. Note that for the BDI, N ≠ 39 in the analysis (cycle 1: *N* = 36) because some participants were only included in subsequent cycles, while others did not complete all cycles (applies for GAD-7, as well).

**Table 2:** Descriptive statistics and ANOVA of the BDI, BSL-23, and GAD-7.

	t0	t1	t2	t0	t1	t2	t0	t1	t2	t0	t1	t2	t0	t1	t2
**BDI**															
N	36	34	24	21	14	15	13	7	7	8	4	4	4	3	2
Mean	29.6	21.3	18.2	19.1	10.5	13.5	25.4	20.8	13.1	20.5	12.5	13.0	16.7	10.0	16.5
SD	11.7	9.9	10.2	9.2	5.9	8.1	11.3	9.5	9.4	7.0	6.4	9.2	8.1	5.3	12.0
	F(2,49) = 16.5, *p* < 0.001, ηp2 = 0.452; *N* = 21			F(1.2,8.5) = 2.95, *p* = 0.118, ηp2 = 0.297; *N* = 8											
**BSL-23**															
N	13	13	8	8	7	4	7	7	1	2	2	0	2	2	1
Mean	42.8	30.9	25.6	32.1	17.6	25.3	48.9	29.0	32.0	41.0	38.0	0	36	17	32
SD	19.5	15.3	14.5	26.0	16.9	18.9	8.3	16.1	0	29.7	15.6	0	15.6	24	0
	F(2,14) = 6.16, *p* = 0.012, ηp2 = 0.468; *N* = 8														
**GAD-7**															
N	9	9	7	6	3	4	3	2	2	2	0	2			
Mean	15	14.2	13	17.8	11.3	14	15	18	13	13	0	13			
SD	3.6	3.5	4.6	2.7	6.7	5.7	4.6	2.1	5	2.8	0	4.3			

	**Cycle 1**			**Cycle 2**			**Cycle 3**			**Cycle 4**			**Cycle 5**		
	t0	t1	t2	t0	t1	t2	t0	t1	t2	t0	t1	t2	t0	t1	t2
**BDI**															
N	36	34	24	21	14	15	13	7	7	8	4	4	4	3	2
Mean	29.6	21.3	18.2	19.1	10.5	13.5	25.4	20.8	13.1	20.5	12.5	13.0	16.7	10.0	16.5
SD	11.7	9.9	10.2	9.2	5.9	8.1	11.3	9.5	9.4	7.0	6.4	9.2	8.1	5.3	12.0
	F(2,49) = 16.5, *p* < 0.001, ηp2 = 0.452; *N* = 21			F(1.2,8.5) = 2.95, *p* = 0.118, ηp2 = 0.297; *N* = 8											
**BSL-23**															
N	13	13	8	8	7	4	7	7	1	2	2	0	2	2	1
Mean	42.8	30.9	25.6	32.1	17.6	25.3	48.9	29.0	32.0	41.0	38.0	0	36	17	32
SD	19.5	15.3	14.5	26.0	16.9	18.9	8.3	16.1	0	29.7	15.6	0	15.6	24	0
	F(2,14) = 6.16, *p* = 0.012, ηp2 = 0.468; *N* = 8														
**GAD-7**															
N	9	9	7	6	3	4	3	2	2	2	0	2			
Mean	15	14.2	13	17.8	11.3	14	15	18	13	13	0	13			
SD	3.6	3.5	4.6	2.7	6.7	5.7	4.6	2.1	5	2.8	0	4.3			

Treatment results were compared using rmANOVA. Missing data were deleted listwise. Due to violations of the sphericity assumption, a Greenhouse–Geisser correction was applied to the BDI cycle 2 analysis. Cycles 3 to 5 for the BDI and cycles 2 to 5 for BSL-23 and GAD were not analyzed due to low case count.

**Table 3:** Descriptive statistics and ANOVA of UF (“upper face”) and ULF (“upper and lower face”).

	t0c1	t1c1	t2c1	t0	t1	t2	t0	t1	t2	t0	t1	t2	t0	t1	t2
**BDI-UF**															
N	26	24	16	13	9	8	6	4	3	2	1	2	1	1	1
Mean	29.3	22.7	19.4	16.9	11.9	15.1	29.7	24.8	17	21.5	8	14	12	12	8
SD	11.1	8.9	9.6	6.5	6.3	8.9	8.2	8.7	12.2	6.4		14.1	/	/	/
	F(1.4,16.7) = 9,79, *p* = 0.003, ηp2 = 0.449; *N* = 13														
	t0c1	t1c1	t2c1	t0	t1	t2	t0	t1	t2	t0	t1	t2	t0	t1	t2
**BDI-ULF**															
N	10	10	8	8	5	7	4	2	4	4	3	2	2	2	1
Mean	30.4	17.9	15.8	22.5	8	11.7	19	13	10.3	20	14	12	19	9	25
SD	13.6	11.8	11.7	12	6.7	6.4	11.9	5.7	7.3	7.1	6.9	7.1	9.9	7.1	/
	F(2,14) = 6,74, *p* = 0.009, ηp2 = 0.490; *N* = 8														

Treatment results were compared using rmANOVA. Missing data were deleted listwise. Due to violations of the sphericity assumption, a Greenhouse–Geisser correction was applied to the BDI cycle 1 analysis. Cycles 2 to 5 were not analyzed due to low case count.

## Data Availability

The data presented in this study are available on request from the corresponding author. The data are not publicly available because the primary data are non-anonymous data from the outpatient clinic.
